# Quality of life and depression in patients with amyotrophic lateral sclerosis – does the country of origin matter?

**DOI:** 10.1186/s12904-023-01189-2

**Published:** 2023-06-13

**Authors:** Katarzyna Ciećwierska, Dorothée Lulé, Maksymilian Bielecki, Olga Helczyk, Anna Maksymowicz-Śliwińska, Julia Finsel, Krzysztof Nieporęcki, Peter M. Andersen, Albert C. Ludolph, Magdalena Kuźma-Kozakiewicz

**Affiliations:** 1 Department of Neurology, University Clinical Center of Medical University of Warsaw, Warsaw, Poland; 2 Department of Neurology, University of Ulm, Ulm, Germany; 3 Department of Psychology, SWPS University of Social Sciences and Humanities, Warsaw, Poland; 4 Institute of Clinical Sciences, Neuroscience, Umeå University, Umeå, Sweden; 5 Department of Neurology, Medical University of Warsaw, Warsaw, Poland; 6 Neurodegenerative Diseases Research Group, Medical University of Warsaw, Warsaw, Poland

**Keywords:** Amyotrophic lateral sclerosis, Wellbeing, Quality of life, Depression, Pain

## Abstract

**Background:**

Given the inevitable relentless progressing nature of amyotrophic lateral sclerosis (ALS), it is essential to identify factors influencing patients’ wellbeing. The study aimed to prospectively assess factors influencing the quality of life (QoL) and depression in ALS patients compared to healthy controls (HCs) from Poland, Germany and Sweden and their relationship to socio-demographic and clinical factors.

**Methods:**

314 ALS patients (120 from Poland, 140 from Germany, 54 from Sweden) and 311 age-, sex- and education-level-matched HCs underwent standardized interviews for quality of life, depression, functional status and pain.

**Results:**

Patients from all three countries showed similar levels of functional impairment (ALSFRS-R). Overall, ALS patients assessed their quality of life as lower compared to HCs (p < 0.001 for the anamnestic comparative self-assessment (ACSA), p = 0.002 for the Schedule for the evaluation of the subjective quality of life - SEIQoL- direct weighting (SEIQoL-DW). Also, the German and Swedish patients, but not the Polish, reported higher depression levels than the corresponding HCs (p < 0.001). Analysis of ALS groups revealed that functional impairment was related to a lower quality of life (ACSA) and higher depression levels among German ALS patients. Longer time since diagnosis predicted lower depression and (in male subjects) higher quality of life.

**Conclusions:**

ALS patients assess their quality of life and mood lower than healthy individuals within the studied countries. The relationships between clinical and demographic factors are moderated by country of provenance, which bears implications for the design and interpretation of scientific and clinical studies, which should reflect the complexity and heterogeneity of mechanisms determining QoL.

**Supplementary Information:**

The online version contains supplementary material available at 10.1186/s12904-023-01189-2.

## Background

Amyotrophic lateral sclerosis is a progressive disease that involves degeneration of the central and peripheral motor system [28] leading finally to quadriplegia, anartria, aphagia and respiratory failure [20]. The course of the disorder is inauspicious with 50% of patients dying within three years of onset [28] but up to 10% surviving for over 10 yearswithout intervention [16, 20]. In the terminal stage, patients may develop locked-in syndrome (LIS) characterized by total loss of movements and communication with preserved consciousness [23]. Currently, approved neuroprotective interventions are limited to riluzole and edaravone, both with modest clinical effect [41, 50]. A combination of sodium phenylbutyrate and tauroursodeoxycholic acid (TUDCA) was recently approved in Canada based on data from the CENTAUR phase-2 study [18]. A phase 3 study PHOENIX of the above combination is presently on-going [43]. As the disease progresses, all patients face a number of difficult decisions to make [5], including for life-extending interventions like non-invasive or invasive ventilatory support, placement of a gastro-feeding tube to secure nutrition. A wide range of support equipment is frequently necessary to enable the patient to stay at home (and not move to a nursing facility) and preserve some degree of communication (e.g. eye tracking communication devices) even in patients with a very advanced disease [4]. A variety of drugs are frequently needed to reduce affective lability, anxiety, depression, nausea, dyspnea and/or pain [4]. For the lack of effective, disease-arresting interventions,the palliative care of the ALS patient and their relatives is symptomatic,with a focus on optimizing personal well-being. Its predictors analyzed to date include clinical state, comorbidities, pain, subjective quality of life, depression, demographic factors (sex, education, employment status), religion and spiritual needs [11, 27, 33, 38]. We here hypothesize that the country of provenance, with its cultural/religious characteristics and dominant value systems might co-determine the quality of life and depression levels in patients with ALS. Hence we prospectively assess the quality of life and depression in patients with ALS and matched healthy controls from threewidely different European countries (Poland, Germany, Sweden) and the relationship of these indices with socio-demographic and clinical factors. The three countries lay in a close geographic proximity in the Northern part of Europe but differed in historically-determined political situation and dominant socio-religious background (Sweden being liberal and historically protestant, Poland generally traditional and catholic and Germany, both).

## Methods

### Patients

Three hundred and fourteen ALS patients were consecutively recruited at university in- and out-patient clinics in Warsaw (n = 120), Ulm (n = 140) and Umea (n = 54) prior to the COVID-19 pandemics. They constituted approximately 60–80% of all consecutive patients consulted fulfilling the inclusion criteria at these centers. The most frequent reasons for not participating in the study were patients’ limited time for a visit due to a long commute, mental fatigue, advanced physical impairment and/or lack of informed consent.

The inclusion criteria were: age > 18 years, clinically definite, probable or probable laboratory-supported ALS according to the El Escorial revised criteria [9], sporadic or familial ALS, time since diagnosisof at least 3 months and Polish/German/Swedish mother tongue. Participants with major cognitive impairment and other neurological, psychiatric or general conditions that could (physically or psychologically) potentially influence the study outcome (e.g., diabetes mellitus, stroke or bipolar disease) were excluded from the sample. The healthy control group (HC) consisted of 311 age-, gender-, and education-matched individuals from the three respective countries (n = 100, n = 100 and n = 111) without the diagnosis of neurodegenerative or psychiatric condition. The patients werecompared for age, sex, education and functional status.

### Data collection

The study procedures were performed by neurologists specialized in neuromuscular diseases and certified clinical psychologists. The questionnaires and interviews were conducted in patients’ native languages.

### Clinical data

Functional/clinical status was assessed with the ALS Functional Rating Scale revised form (ALSFRS-R), with results ranging from 0 (locked-in state) to 48 (no functional impairment) [10]. The clinical assessment included the disease duration (time from first definite paretic disease symptom to the day of the survey), the time from diagnosis (time interval from the diagnosis of ALS to the day of the survey) in months, the possible use of a gastric feeding tube (percutaneous endoscopic gastrostomy - PEG or radiologically inserted gastrostomy RIG), invasive (IV) and non-invasive ventilation (NIV).

### Quality of life and depression measures

The measure of global QoL was done with the anamnestic comparative self-assessment (ACSA), which allows estimating current overall well-being on a -5 to 5 scale with the extremes anchored at the memory of the worst (-5) and best (5) periods in the subject’s life [7, 8]. Subjective QoL measurement was obtained using the Schedule for the evaluation of the subjective quality of life - SEIQoL- direct weighting (SEIQoL-DW) [19, 30]. This tool first allows subjects to indicate the five most important areas of their lives. This step is followed by an assessment of the subjective importance and satisfaction related to each of the areas. The final numeric score of SEIQoL-DW is expressed on a 0 to 100 scale [19].

Finally, depressiveness was measured with the ALS-Depression-Inventory 12 Items (ADI-12). Numeric scores on that scale range from 12 to 48 with higher results indicating lower mood levels [17].

For the lack of validated Polish and Swedish translations and for the purpose of the study, all three tools were adapted from the original English versions. To ensure high levels of accuracy and content validity, in each language the same procedure was followed, including translation, independent back-translation, and final verification performed by experienced clinical psychologists fluent in English and the required target language. For the German versions, the existing previously validated translations were used.

Regarding the psychometric properties, neither ACSA (as a single-item measure) nor SEIQoL-DW (with its idiographic approach) allows for the assessment of internal consistency, however, both tools were extensively used in the studies of severely ill populations and have satisfactory psychometric properties, for more in-depth analyses: [44] and [7]. The reliability of ADI-12 was satisfactory in all three tested groups (Cronbach alpha = 0.80, 0.91 and 0.89 for Polish, Swedish and German participants, respectively).

### Pain

The pain was expressed on a quantitative scale (frequency of occurrence pain: How often do you feel pain? 1 = never to 6 = every day; the intensity of pain: How severe is this pain? 1 = lack of pain to 6 = unbearable pain).

### Statistical analysis

Continuous variables are reported as means and standard deviations and discrete variables as frequencies and percentages. Two-way ANOVA with bootstrapped p-values (2000 samples) and logistic regression were used to assess the effects of Country (Poland vs. Germany vs. Sweden) and ALS Diagnosis (ALS patients vs. healthy controls) in explaining group differences observed for continuous and dichotomous dependent variables. Main effects in ANOVA were interpreted using Tukey post-hoc tests and interactions by computing simple effects of Diagnosis within each country.

Comparisons of clinical characteristics across ALS groups were conducted using one-way ANOVA (with Tukey post-hocs) and Chi^2^ test (with Bonferroni-corrected post-hocs).

Additionally, the key quality of life indicators (ACSA, SEIQoL-DW) and depression (ADI) were tested using multivariate regression including all main effects and two-way interactions of predictors. To facilitate interpretation of the coefficients, categorical variables were contrast coded and continuous predictors – mean-centered prior to analysis. P-values < 0.05 were considered statistically significant. Computations were conducted using IBM SPSS (version 25) and R version 4.0.2 [35].

### Power and sensitivity calculation

The study was a part of the JPND funded scientific project „NEEDS in ALS” [48] focusing on the end-of-life decisions of patients with ALS. Details of the initial power calculations specific to the primary goals of the project (not directly related to this publication) are available in [5].

Considering the aims and design of the study, we initially performed sensitivity analysis using G*POWER (ver. 3.1, [13]). These showed that - given available sample size and an alpha level of 0.05 - our design offered sufficient power to detect small to small-medium effects (as defined using conventional cut-offs, see: [12]). More specifically, 80% power in 2-way ANOVA was obtained for *η*_*p*_^*2*^ = 0.01 (main effect of diagnosis) and *η*_*p*_^*2*^ = 0.02 (main effect of country and country x diagnosis interaction), in 1-way ANOVA (ALS groups) for *η*_*p*_^*2*^ = 0.03 effects, and, in the regression models, for *f*^*2*^ = 0.07 (tests of single predictors).

## Results

### Demographic characteristics

No significant effects were observed in the distribution of gender across groups (see Table 1, which summarizes the demographic characteristics of ALS patients and control cohorts, for all the descriptive statistics). ALS patients were slightly older than controls (p = 0.021). Polish individuals were younger than Swedish and German subjects (p = 0.007) and reported a higher level of education compared to the Germans cohorts (p = 0.005). For the Swedish cohort, the control group had a higher level of education than the ALS patients groups (Country x Diagnosis interaction, p = 0.005).

**Table 1:** Descriptive statistics and between-group comparisons of the demographic and clinical characteristics

	N						Mean						Standard Deviations						
	Germany		Poland		Sweden		Germany		Poland		Sweden		Germany		Poland		Sweden		
	ALS	HCs	ALS	HCs	ALS	HCs	ALS	HCs	ALS	HCs	ALS	HCs	ALS	HCs	ALS	HCs	ALS	HCs	Significant effects in Country x Group ANOVA^+^
**Age (years)**	140	100	120	100	54	111	62.8	60.1	59.4	59.7	65.6	61.3	11.2	6.2	11.7	13.3	10.0	14.3	**Group** (0.021): ALS > HC, **Country** (0.007): SE > PL, DE > PL
**Education (years)**	140	99	111	100	47	111	13.2	12.8	13.9	13.8	12.1	13.8	3.0	2.8	3.8	3.1	3.0	2.8	**Country** (0.005): PL > DE, **Interaction** (0.005): SE: HC > ALS
**QoL: ACSA**	138	100	117	100	51	111	-0.3	1.5	-0.2	1.2	0.2	2.6	2.6	1.6	2.6	1.7	2.7	1.6	**Group** (< 0.001): Contr > ALS; **Country** (< 0.001): SE > PL, SE > DE
**QoL: SEIQoL-DW**	132	96	115	100	50	110	72.5	74.1	65.9	72.5	75.4	79.0	16.0	11.3	18.6	15.0	18.3	10.8	**Group** (0.002): Contr > HC; **Country** (< 0.001): SE > DE > PL
**Pain frequency**	126	100	102	100	24	111	3.3	2.9	3.4	3.7	3.6	2.9	2.2	1.6	2.3	1.7	2.4	2.2	**Country** (0.043): PL > DE, PL > SE
**Pain intensity**	125	100	102	100	19	111	2.2	2.3	2.2	2.6	2.6	2.1	1.3	1.1	1.3	0.8	1.3	1.3	**Interaction** (0.020): in PL HC > ALS
**ADI-12**	137	100	110	100	54	111	24.7	18.5	25.2	23.9	21.4	16.4	6.6	4.6	5.1	4.8	6.1	3.9	**Group** (< 0.001): ALS > HC; **Country**(< 0.001): PL > DE > SE; **Interaction** (< 0.001): in SE: ALS > HC, in DE: ALS > HC
	**N (total)**						**N (males)**						**% (males)**						**Country x Group Logistic regression**
**Sex**	140	100	120	100	54	111	66	52	55	50	30	71	**47.1**	52.0	**45.8**	50.0	**55.6**	64.0	no significant effects

*Note*: ALS: amyotrophic lateral sclerosis, HCs: healthy controls; QoL: quality of life; ALSFRS-R: amyotrophic lateral sclerosis functional rating scale-revised; ACSA: anamnestic comparative self-assessment; SEIQoL-DW: Schedule for the evaluation of the subjective quality of life - SEIQoL- direct weighting; ADI-12: ALS-Depression-Inventory 12 Items

^*+*^ Direction and significance of all effects in 3 × 2 between-subject ANOVAs with bootstrap p-values. All significant interactions and main effects are reported with accompanying p-values. Main effects of Country are interpreted by reporting the patterns of significant Tukey post-hoc comparisons. Interactions are interpreted by reporting significant simple effects of Group for each country

### Clinical characteristics of the three ALS cohorts

There were significant differences between countries in the disease duration (p < 0.001) and time since diagnosis (p < 0.001). The highest means were observed in the Swedish cohort, followed by the Polish and German groups (detailed statistics are presented in Table 2, which summarizes the clinical characteristics of patients and controls). Despite varied disease duration, no significant differences in ALSFRS-R scores (p = 0.375) or progression rate (p = 0.098) at the time of evaluation were observed. Also, the proportion of patients with bulbar vs. spinal onset was similar across groups (p = 0.098).

**Table 2:** Comparison of clinical characteristics of German, Polish, and Swedish samples of ALS patients

	ALS Germany			ALS Poland			ALS Sweden				
	N	M	SD	N	M	SD	N	M	SD	p-value	post-hoc tests
**disease duration (months)**	137	**22.91**	26.34	115	**26.77**	11.37	51	**34.61**	14.14	**< 0.001**	SE > PL > DE
**time since diagnosis (months)**	137	**11.04**	12.40	115	**11.63**	7.13	51	**17.82**	9.00	**< 0.001**	SE > PL = DE
**ALSFRS-R**	137	**34.48**	34.39	115	**34.87**	8.09	51	**33.04**	6.42	0.375	
**Progression rate** ^**+**^	137	**0.69**	0.48	115	**0.65**	0.52	51	**0.52**	0.37	0.098	
	** N (total)**	N	**%**	**N (total)**	**N**	**%**	**N (total)**	**N**	**%**	**p-value**	**post-hoc tests**
**bulbar (vs. spinal) onset**	140	40	**28.6**	117	27	**23.1**	53	13	**24.5**	0.589	
**PEG/RIG**	140	17	**12.1**	116	6	**5.2**	53	13	**24.5**	**0.001**	SE > PL
**NIV**	140	50	**35.7**	116	7	**6.0**	53	13	**24.5**	**< 0.001**	SE = DE > PL

*Note*: PEG: percutaneous endoscopic gastrostomy; RIG: radiologically inserted gastrostomy; NIV: non-invasive ventilation

^+^Progression rate: ALSFRS points per month since onset

### Quality of life and depression measures

**Fig. 1:**
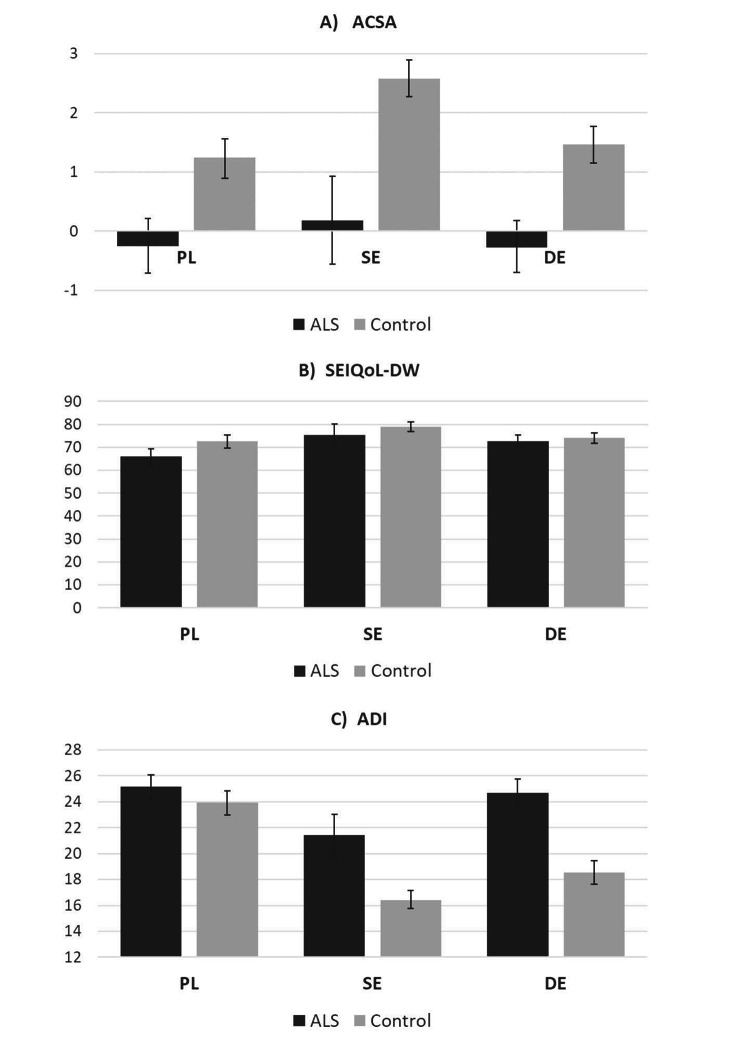
Mean scores of ACSA (a), SEIQoL-DW (b), and ADI (c) across studied groups. Error bars depict 95% bootstrap confidence intervals *Note*: ACSA: anamnestic comparative self-assessment; SEIQoL-DW: Schedule for the evaluation of the subjective quality of life - SEIQoL- direct weighting; ADI: ALS-Depression-Inventory 12 Items

The two-way ANOVA revealed a similar pattern of results for all three key measures (effect of illness, effect of country and country x diagnosis interaction). The quality of life in ALS patients was lower (p < 0.001 for ACSA, p = 0.002 for SEIQoL-DW) and depression levels higher compared to control groups (p < 0.001, see Table 1; Fig. 1 for descriptive statistics). Significant effects of country were revealed for all measures (each p < 0.001) with the Swedish sample reporting higher quality of life and lower depression level than the two other groups, and Polish participants obtaining lower SEIQoL-DW and higher depression scores when compared with other countries. Noticeably, a significant country x diagnosis interaction was observed for ADI scores, with ALS groups scoring on average higher than controls in both Germany and Sweden, but not in Poland.

### Pain

Polish HCs assessed their severity of pain as higher compared to ALS patients (p = 0.020). There were no significant differences in severity and frequency of pain between patients and controls in the other groups. A significant effect of country was revealed for frequency of pain (p = 0.043) with Polish patients reporting higher frequency than German and Swedish patients. There was no significant difference in the severity of pain between countries.

### Multiple regression models in the ALS cohorts

A series of multiple regression models were fitted for each of the key indicators (ACSA, SEIQoL-DW and depression). For each model, the same set of predictors was used: country, age (in years), months since disease onset (disease duration) and gender. The regression model included the main effects and all two-way interactions of the predictors listed above, hence obtained differences might be interpreted as adjusted for demographic and clinical variables (significance of the tested effects is reported in Table 3, complete set of estimates in Table 4).

**Table 3:** Summaries of the multiple regression models predicting ACSA, SEIQoL-DW and ADI scores in ALS groups. Significant effects are printed in bold

	ACSA			SEIQoL-DW			ADI-12		
	df	F	p	df	F	p	df	F	p
**Country**	2	0.15	0.859	2	7.30	**0.001**	2	6.55	**0.002**
**Age**	1	0.18	0.675	1	0.06	0.810	1	3.72	0.055
**Sex**	1	1.22	0.271	1	1.41	0.235	1	3.04	0.082
**ALSFRS-R**	1	5.07	**0.025**	1	1.47	0.226	1	20.03	**< 0.001**
**Disease duration**	1	2.76	0.098	1	0.33	0.563	1	8.27	**0.004**
**Country x Age**	2	0.45	0.640	2	1.33	0.265	2	0.47	0.627
**Country x Sex**	2	1.75	0.175	2	1.72	0.182	2	0.94	0.392
**Country x ALSFRS-R**	2	5.74	**0.004**	2	2.58	0.077	2	3.71	**0.026**
**Country x Disease duration**	2	0.36	0.697	2	0.69	0.501	2	0.28	0.756
**Age x Gender**	1	0.11	0.738	1	0.59	0.441	1	3.19	0.075
**Age x ALSFRS-R**	1	3.10	0.079	1	0.11	0.741	1	0.20	0.658
**Age x Disease duration**	1	0.01	0.923	1	0.01	0.936	1	0.43	0.512
**Gender x ALSFRS-R**	1	0.02	0.875	1	1.98	0.160	1	2.23	0.137
**Gender x Disease duration**	1	4.01	**0.046**	1	0.12	0.730	1	0.01	0.904
**ALSFRS-R x Disease duration**	1	0.18	0.675	1	0.41	0.524	1	0.74	0.390
**Model statistics**	*F*(20, 279) = 2.20, *p* = 0.002, *R*^*2*^ = 0.136, Adjusted *R*^*2*^ = 0.074			* F*(20, 269) = 1.78, *p* = 0.023, *R*^*2*^ = 0.117, Adjusted *R*^*2*^ = 0.051			* F*(20, 273) = 4.56, *p* < 0.001, *R*^*2*^ = 0.251, Adjusted *R*^*2*^ = 0.196		

*Note*: ALSFRS-R: amyotrophic lateral sclerosis functional rating scale-revised. ACSA: anamnestic comparative self-assessment; SEIQoL-DW: Schedule for the evaluation of the subjective quality of life - SEIQoL- direct weighting; ADI-12: ALS-Depression-Inventory 12 Items

**Table 4:** Regression coefficients obtained in multiple regression models predicting ACSA, SEIQoL-DW and ADI results in ALS patients

	ACSA		SEIQoL-DW		ADI-12	
Term	b	95% CI	b	95% CI	b	95% CI
**(Intercept)**	-0.19	[-0.57, 0.18]	70.54**	[67.90, 73.17]	23.88**	[23.08, 24.69]
**Country 1**	0.05	[-0.57, 0.67]	5.27*	[0.87, 9.67]	-2.28**	[-3.61, -0.94]
**Country 2**	0.06	[-0.38, 0.51]	43,891	[-2.07, 4.14]	0.56	[-0.40, 1.53]
**Age**	0.01	[-0.02, 0.04]	0.03	[-0.20, 0.26]	0.07	[-0.00, 0.14]
**Gender**	0.19	[-0.15, 0.52]	15,342	[-0.93, 3.76]	-0.64	[-1.36, 0.08]
**ALSFRS-R**	0.05*	[0.01, 0.09]	0.19	[-0.12, 0.49]	-0.20**	[-0.29, -0.11]
**Disease duration**	0.02	[-0.00, 0.04]	0.05	[-0.11, 0.20]	-0.07**	[-0.12, -0.02]
Country 1 * Age	0.01	[-0.05, 0.06]	0.01	[-0.39, 0.41]	0.06	[-0.06, 0.18]
Country 2 * Age	-0.02	[-0.06, 0.02]	0.17	[-0.12, 0.46]	-0.03	[-0.12, 0.06]
Country 1 * Gender	-0.15	[-0.71, 0.40]	22,706	[-0.26, 7.51]	0.81	[-0.39, 2.01]
Country 2 * Gender	-0.24	[-0.67, 0.19]	-1.99	[-5.02, 1.04]	-0.25	[-1.18, 0.69]
Country 1 * ALSFRS-R	-0.04	[-0.10, 0.02]	-0.20	[-0.67, 0.27]	0.05	[-0.09, 0.18]
Country 2 * ALSFRS-R	0.09**	[0.04, 0.14]	0.45*	[0.06, 0.83]	-0.16**	[-0.27, -0.04]
Country 1 * Disease duration	0.01	[-0.02, 0.04]	-0.09	[-0.31, 0.14]	-0.01	[-0.08, 0.06]
Country 2 *Disease duration	0.00	[-0.03, 0.04]	-0.04	[-0.26, 0.18]	-0.01	[-0.08, 0.06]
Age * Gender	0.00	[-0.02, 0.03]	0.08	[-0.12, 0.28]	-0.06	[-0.12, 0.01]
Age * ALSFRS-R	0.00	[-0.00, 0.01]	0.00	[-0.02, 0.03]	-0.00	[-0.01, 0.01]
Age * Disease duration	0.00	[-0.00, 0.00]	0.00	[-0.02, 0.02]	-0.00	[-0.01, 0.00]
Gender * ALSFRS-R	0.00	[-0.04, 0.04]	0.21	[-0.08, 0.51]	-0.06	[-0.15, 0.02]
Gender * Disease duration	-0.02*	[-0.04, -0.00]	-0.03	[-0.18, 0.13]	0.00	[-0.05, 0.05]
ALSFRS * Disease duration	0.00	[-0.00, 0.00]	-0.01	[-0.03, 0.01]	0.00	[-0.00, 0.01]

*Note*: Disease duration: time since disease onset (in months); ALSFRS-R: amyotrophic lateral sclerosis functional rating scale-revised; ACSA: anamnestic comparative self-assessment; SEIQoL-DW: Schedule for the evaluation of the subjective quality of life - SEIQoL- direct weighting; ADI-12: ALS-Depression-Inventory 12 Items. Countries are represented using contrasts codes: Country 1 (SE - PL), Country 2 (DE - PL). Contrast coding for Gender: Females = 1, Males = -1. All continuous predictors are mean-centered

* indicates p < 0.05; ** indicates p < 0.01

Analysis of ACSA scores revealed a significant main effect of the functional state: higher scores of ALSFRS corresponded to a higher quality of life (b = 0.05, p = 0.025). This relationship was moderated by the effects of the Country (p = 0.004 for the interaction effect, Fig. 1A). Simple slopes analysis showed that the ALSFRS-R - ACSA relationship was significant only in the German patients (b = 0.14, p < 0.001), whereas in Poland (b = -0.01, p = 0.901) and Sweden (b = 0.01, p = 0.848) no such effect was observed. Furthermore, a marginally significant association between sex and disease duration was observed (p = 0.046, Fig. 1B). In female participants there were no significant changes in ACSA related to the disease duration (b = 0.00, p = 0.790), while in male patients a longer disease duration was related to higher subjective life quality (b = 0.041, p = 0.013).

The model focused on SEIQoL-DW revealed a significant effect of the Country (p = 0.001). Pairwise comparisons showed that Polish participants declared a significantly lower quality of life (M = 64.2) when compared both with German (M = 71.8, p = 0.004) and Swedish participants (M = 75.8, p = 0.004).

Analysis of ADI scores showed that lower ALSFRS-R scores (b = -0.20, p < 0.001) and shorter disease duration (b = -0.07, p = 0.004) were independently both related to higher level of depression. A significant effect of the country (p = 0.002) could be explained by lower depression scores observed in Swedish patients (M = 21.43) when compared with Polish (M = 25.17, p = 0.001) and German (M = 24.68, p = 0.022) participants. The last significant effect, the interaction of ALSFRS-R and the country (p = 0,026, Fig. 1C), closely mirrored effects observed for ACSA scores. ALSFRS-R was not significantly related to declared depression in Polish (b = − 0.09, p = 0.31) and Swedish (b = -0.16, p = 0.06) patients, while in German patients a lower ALSFRS-R score predicted a significantly higher depression levels (b = -0.36, p < 0.001). Due to a low number of patients using PEG or NIV/IV, these variables could not be included as predictors in the regression analyses. Partial correlations between PEG and NIV presence and quality of life measures controlling for the effects of sex, age, ALS-FRS results, and time since ALS onset showed no significant effects (all uncorrected p/values > 0.1; see Suppl. Material).

## Discussion

Subjective life satisfaction is conditioned by a combination of physical, psychological, social and environmental factors. The determinants of wellbeing in ALS exceed medical and physical domains. For a progressive and irreversible loss of physical autonomy along the disease course, social, intellectual and spiritual factors may have a more prominent role [11, 14, 40]. We hypothesized that, in this context, both socio-economical and socio-cultural factors differentiating studied countries might significantly shape patients’ wellbeing.

### Comparisons of ALS patients and healthy controls

Not surprisingly, the ALS patients in all three countries (n = 314) assessed their quality of life as lower than healthy individuals. Similar effects have previously been reported among German ALS patients at with more advanced disease stage and a longer duration (n = 159) [21] but not in an other smaller study cohorts [22, 24]. Furthermore, despite apparent differences between countries, neither of the two analyses performed on QoL measures revealed any interaction effects, suggesting that the negative impact of ALS on QoL is comparable across studied populations. This pattern of intercountry differences is not specific to our results and closely matches the results observed in many large-scale studies performed on representative samples, including our previous report [5]. Relatively highest levels of wellbeing indices are regularly reported in Sweden, followed by the results from Germany, and lowest scores from Poland (see: results of the Third European Quality of Life Survey [1]).

The coherent picture of the effects of diagnosis and country being additive, becomes more complex when contrasted with the results observed for depression, where significant effects of ALS could be observed only in Sweden and Germany, but not in the Polish group. This surprising finding seems to be driven mainly by unusually high depression scores in the Polish control group. Similarly, the analysis of pain intensity showed a paradoxical effect of significantly higher pain levels in healthy Polish controls when compared with ALS patients. Both these effects might be to some extent interpreted as symptoms of a culturally specific phenomenon known as the Polish culture of complaining [45]. From a broader methodological perspective, it illustrates that - even within the same country, language, and culture - the invariance of scores might be difficult to achieve.

#### Predictors of QoL and depression in ALS patients

In the second step of the analysis, the use of multivariate regression allowed us to disentangle more universal relationships from country-specific effects and better understand the contribution of clinical and demographic predictors in explaining the QoL and depression levels. However, before discussing the detailed results, it is essential to emphasize that we describe a multivariate model adjusted for the differences in scores across countries. This strategy allows us to interpret the effects of other predictors more confidently without assuming full invariance of scores among countries/language versions.

We start the discussion by addressing one of the ongoing debates in ALS literature - the potential significance of functional state in explaining patients’ psychological wellbeing. The association between a higher quality of life and patients’ disease progression was reported in single- (n = 330) and multi-center (n = 325) studies conducted in Germany [34, 49], as well as Iranian (n = 132) and Brazilian (n = 45) cohorts [3, 39]. On the other hand, in an American study (n = 62) Robbins et al. showed that the QoL was not determined by physical decline in general [37]. Similar incoherence was observed in studies focused on depression levels. Poor physical state correlated with higher depression levels (as measured using Beck Depression Inventory) [21] in German patients. At the same time, no such correlations were observed in smaller samples from the US (n = 56 and n = 36, respectively) [32, 36] and Germany [24]. Our results contribute to that discussion by revealing a considerable heterogeneity of effects across countries. In our case, results from Poland and Sweden suggest no relationship between the functional state and depression or quality of life measured by ACSA, and results from Germany showing significant negative psychological consequences of ALS progression. To the best of our knowledge, no such results were ever reported within a single, large scale and multinational study.

Considering a broad range of predictors and their interactions, we could contribute to a discussion on a second contentious issue - the adaptation process following ALS diagnosis. That is also an area of research far from reaching a consensus. Earlier American [6] and German [21] studies carried out in patients at a similar or more advanced disease stage did not detect any correlations between the disease duration and depression levels. However, prospective studies have revealed that ALS patients’ desire to hasten death decreased significantly within a year of diagnosis, despite progressive physical function decline [25]. It supports the existence of one or more efficient adaptation mechanism. In our analysis of depression levels, a longer disease duration was predictive of a relatively better mood (after controlling for all the covariates). These results are coherent with more recent studies [22, 26]. At the same time, a similar adaptive mechanism was observed in our analysis of the subjective QoL, but the disease length’s effect was this time limited to male patients only. This findings again points to the vital role of moderators and the complexity of the studied phenomena.

Finally, as our study was conducted simultaneously in three large cohorts, it might also be worth mentioning one of the negative results of the statistical analyses, namely the lack of statistically significant effects of age. Age is recognized to influence the QoL and depression measures in the general population [2, 42]. In ALS, it was shown to be related to specific coping strategies [31] and likely to influence processes of decision-making related to care [15]. In the specific context of ALS, the wellbeing mainly depended on efficient individual coping strategies and velocity of disease progression [24], rather than processes significantly determined by age (or its correlates), as shown in our cohort.

To summarize, we believe that our study provides a cautionary tale by showing the intrinsic complexity and heterogeneity of the mechanism determining patients’ psychological wellbeing. The set of factors determining QoL or depression levels seems to significantly vary between countries, which should be taken into account both in the interpretation of the existing literature, as well as in designing future research (including international clinical trials in ALS, where the changes in QoL and depression following the pharmacological intervention are frequently secondary end-points) [46, 47, 29 and others].

## Study limitations

1) The patients who participated in the study were followed at specialized multidisciplinary care centers, which might have influenced their overall well-being.

2) Some of the Swedish patients who were recruited to the study had a longer disease duration and comparable functional impairment implicating a more benign disease course and longer adaptation period.

3) The regression approach adjusted for the non-equality of mean scores between ALS groups. However, the interpretability of the regression coefficients of main effects still required metric invariance. Strong violations of that assumption could cast doubt on some of our conclusions.

4) The analysis would be more comprehensive if it considered the socioeconomic background and information about support (e.g. health-system, social support, etc.). Regrettably, such data were not available within this project.

5) The data was gathered prior to the COVID19 pandemic and does not reflect the present (unstable) situation of the healthcare systems in the three countries. At the same time, our results may reflect more stable processes not affected by transient external factors.

## Conclusions

Our results confirmed that ALS patients assess their quality of life as lower and depression levels as higher than healthy individuals. We also demonstrate that both QoL and depression (at least in some studied subgroups of patients) are significantly affected by two opposing forces: the beneficial process of adaptation to the diagnosis, and declining functional state leading to significant psychological costs. Our results also bear methodological implications, pointing to the importance of the use of country-specific control groups, taking into account the heterogeneity of the effects and limited generalizability of the results across cultural contexts (even in relative geographical proximity) at all stages of the research process.

## Electronic supplementary material

Below is the link to the electronic supplementary material.

Supplementary Material 1

## Data Availability

The datasets used and/or analysed during the current study are available from the corresponding author on reasonable request.

## Abbreviations

ALS: Amyotrophic lateral sclerosis, HCs:healthy controls
QoL: Quality of life
ALSFRS-R: Amyotrophic lateral sclerosis functional rating scale-revised
ACSA: Anamnestic comparative self-assessment
SEIQoL-DW: Schedule for the evaluation of the subjective quality of life - SEIQoL- direct weighting
LIS: Locked-in syndrome
TUDCA: Sodium phenylbutyrate and tauroursodeoxycholic acid
PEG: Percutaneous endoscopic gastrostomy
RIG: Radiologically inserted gastrostomy
IV: Invasive ventilation
NIV: Non-invasive ventilation
ADI-12: ALS-Depression-Inventory 12 Items
