# Supplementary material for: Quality of life and depression in patients with amyotrophic lateral sclerosis – does the country of origin matter?
Source: BMC Palliat Care. 2023 Jun 13;22:72. doi: 10.1186/s12904-023-01189-2 (PMC10262126; doi:10.1186/s12904-023-01189-2)
Supplement: Supplementary file 1 — Supplementary Material 1 [file 12904_2023_1189_MOESM1_ESM.pdf]

Quality of life and depression in patients with amyotrophic lateral sclerosis – does the country of origin matter?

Ciećwierska et al., BMC Palliative Care, 2023

Supplementary Materials

Correlations between PEG/RIG and NIV usage and quality of life outcomes

Given the observed frequencies, PEG (percutaneous endoscopic gastrostomy or radiologically inserted gastrostomy – RIG) and NIV (non-invasive ventilation) could not be included in the regression models, as reliable estimation of their effects within each country would not be warranted. Therefore, we have run additional analysis, computing partial correlations between PEG/NIV and critical quality of life outcomes within each country controlling for the same set of predictors as in the regression analysis (age, sex, ALS-FRS, time since onset). None of the observed relationships was significant (all uncorrected *p*-values were above .1).

Table S1. Partial correlations between PEG and NIV presence and quality of life measures controlling for the effects of sex, age, ALS-FRS results, and time since ALS onset.

			**ACSA**	**SEIQoL-DW**	**ADI-12**
**Germany**	**PEG**	**Partial correlation**	**-0.08**	**0.05**	**-0.05**
		Significance	0.345	0.557	0.545
		N	134	127	132
	**NIV**	**Partial correlation**	**-0.04**	**0.12**	**0.02**
		Significance	0.65	0.195	0.801
		N	134	127	132
**Poland**	**PEG**	**Partial correlation**	**-0.17**	**0.05**	**0.14**
		Significance	0.086	0.627	0.166
		N	107	106	101
	**NIV**	**Partial correlation**	**-0.10**	**0.02**	**-0.03**
		Significance	0.291	0.864	0.797
		N	107	106	101
**Sweden**	**PEG**	**Partial correlation**	**0.03**	**0.01**	**0.24**
		Significance	0.85	0.955	0.1
		N	46	45	49
	**NIV**	**Partial correlation**	**0.12**	**0.15**	**0.01**
		Significance	0.439	0.316	0.954
		N	46	45	49

Note: ACSA: anamnestic comparative self-assessment; SEIQoL-DW: Schedule for the evaluation of the subjective quality of life - SEIQoL- direct weighting; ADI-12: ALS-Depression-Inventory 12 Items.
